# Beyond Memory, Learning, and Executive Functions: New Directions for the Development of More Sensitive Measures

**DOI:** 10.1002/alz70857_098432

**Published:** 2025-12-24

**Authors:** Kirsten Horne Crenshaw

**Affiliations:** ^1^ University of Miami, Miami, FL, USA

## Abstract

**Background:**

Semantic Intrusion Errors (SIEs) occur when a person incorrectly produces a word that is semantically related to the intended target. The Loewenstein‐Acevedo Scales for Semantic Interference and Learning (LASSI‐L) is an innovative cognitive challenge test designed to tap SIEs. SIEs has proven to be effective for identifying early cognitive changes associated with both imaging and blood‐based biological markers of Alzheimer's disease (AD) and AD‐related dementias (AD/ADRD). SIEs may reflect executive deficits in self‐monitoring and inhibition with damage of connections between prefrontal and medial temporal circuitry but may also stem from deficits in delayed semantic source memory.

**Method:**

Forty‐five older adults received clinical and cognitive evaluations and deemed cognitively unimpaired (CU) and *p*‐tau217 negative (*n* = 25) or cognitively impaired (CI) *p*‐tau217 positive and met criteria for amnestic mild cognitive impairment (MCI) (*n* = 20). All participants were administered the LASSI‐L with a delayed semantic source memory recognition condition. Multiple linear regressions analyses were performed to examine associations between performance on the LASSI‐L and a ubiquitously used test of executive function, the Wisconsin Card Sorting Test (WCST).

**Results:**

After adjusting for age, MMSE score SIEs on the LASSI‐L, WCST perseverative errors and total correct categories continued to differentiate diagnostic groups. Importantly, perseverative errors on WCST related to SIE's on subtests vulnerable to proactive semantic interference (PSI) (*r* = 0.493, *p* = 0.002) and the failure to recover from PSI (frPSI) (*r* = 0.416, *p* = 0.009). In contrast, only non‐preservative errors were correlated with correct responses on List Cued B1 (*r* = 0.445, *p* = 0.005) and List Cued B2 (*r* = 0.618, *p* < .001) trials (but not SIEs). Moreover, both correct recognition for List A targets (β=‐0.529, *p* < .001) and WCST perseverative errors (β=0.359, *p* = 0.007) were independent predictors of outcome explaining 51.5% of the variance (*R* ^2^ = 0.515).

**Conclusions:**

These findings indicate SIEs on the LASSI‐L reflect both source memory and specific deficits in executive function. LASSI‐L presents as a compelling alternative to traditional memory assessments, and may also be useful for detecting subtle deficits in executive function. Which may be found during the earliest stages of AD/ADRD.

